# The Value of Next-Generation Sequencing for Treatment in Non-Small Cell Lung Cancer Patients: The Observational, Real-World Evidence in China

**DOI:** 10.1155/2020/9387167

**Published:** 2020-01-24

**Authors:** Yan Zhang, Wen-Xiang Shen, Li-Na Zhou, Min Tang, Yue Tan, Chun-Xia Feng, Ping Li, Li-Qiang Wang, Min-Bin Chen

**Affiliations:** Department of Oncology, Affiliated Kunshan Hospital of Jiangsu University, Jiangsu, China

## Abstract

**Background:**

Great success has been made in the targeting therapy of advanced non-small cell lung cancer (NSCLC). Nowadays, next generation sequencing (NGS) is acquirable and affordable in developed area of China. Using this feasible and accurate method of detecting therapeutic genes would help to select optimal treatments to extend patients survival. Here, we identified somatic mutations by NGS and analyzed the value for treatment of NSCLC in a real-world clinical setting.

**Methods:**

NGS was carried out on biopsy samples obtained from 66 advanced unresectable NSCLC patients who had not received any treatment. 23 patients received liquid biopsy after failure of first-line targeted treatment. The mutation profiling as well as associations between mutations and clinicopathological characters was analyzed. The study also assessed the values of NGS for choosing treatment options and predicting prognosis in NSCLC patients.

**Results:**

152 somatic mutations were identified in 45 (68.18%) tissue samples. The most frequently mutated genes were *EGFR* (42.42%), *TP53* (31.82%) and *KRAS* (15.15%). Specifically, the most frequent *EGFR* (42.42%), *EGFR* (42.42%), *p* = 0.046). In addition, in the smoking group, patients with *EGFR* (42.42%), *p* = 0.046). In addition, in the smoking group, patients with *EGFR* (42.42%), *EGFR * (42.42%), *p* = 0.046). In addition, in the smoking group, patients with

**Conclusions:**

The observational study from real-world demonstrated that using NGS in routine clinical detection may be useful in guiding the therapy decisions and benefit more Chinese NSCLC patients.

## 1. Introduction

Non-small cell lung cancer (NSCLC) contributes to over 80% of all lung cancer cases and it is one of the leading causes of cancer-related deaths worldwide [1]. Although the application of surgery, chemotherapy, radiation and targeted therapy was beneficial for some patients, most of patients died of relapse, metastasis or even adverse effects by treatment [2, 3]. Epidermal growth factor receptor (*EGFR*)-tyrosine kinase inhibitors (TKI) have been used to treat NSCLC since 2003 with great success in patients with *EGFR* mutations [4–6], which made physicians pay more attention to “individualized treatment”.

Unfortunately, acquired resistance to EGFR-TKI treatment occurs inevitably. It is well known that NSCLC patients with the *EGFR* exon 19 deletion or L858R mutation show initial responses to first-generation of TKI, such as gefitinib and erlotinib. After about 9–14 months treatment, more than half of the patients relapsed [7]. Possible mechanisms for the acquired resistance may be the appearance of second-site hot spot mutations [8]. The most common and famous alteration is *EGFR* T790M mutation. The efficiency of the third-generation EGFR-TKI osimertinib in treating *EGFR *T790M mutated patients has been demonstrated in several studies [9–11]. However, more targeted and rare mutations and biomarkers are needed to use in clinical practice for prolonging survival of NSCLC patients.

With the wide use of next-generation sequencing (NGS), the genetic basis of various diseases, especially human cancers, have been disclosed. NGS is a high-throughput method that can detect numerous genetic variations, such as single nucleotide variants, insertions and deletions, copy number variations, and gene fusions over larger genomic regions. It is also noted for its high sensitivity and specificity [12]. Consequently, NGS may be a good tool for guiding the treatment of NSCLC.

The aim of this study was to identify somatic mutations by NGS and analyze the value for treatment in NSCLC patients in a real-world clinical setting.

## 2. Materials and Methods

### 2.1. Study Population

This study included 66 histologically confirmed NSCLC cases diagnosed in Affiliated Kunshan Hospital of Jiangsu University between January 2010 and September 2017. They were all advanced unresectable NSCLC patients. Tissues were obtained by transbronchoscopic lung biopsy, lymph node biopsy, thoracentesis or lumbar puncture. The personal data of each participant about clinical characteristics and survival information was collected from clinical record or family contact. The overall survival (OS) was defined as time from the data of diagnosis to the data of death or last visit. The progression-free survival (PFS) was calculated from the time of diagnosis to the time of progression, relapse, death, or the last follow-up. This prospective observational study was reviewed by our institutional review board and written informed consent was provided by each patient.

### 2.2. Tissue DNA and Plasma Cell-Free DNA Extraction

Tissues were stored at −80°C until DNA extraction. Genomic DNA was extracted using a QIAamp DNA FFPE tissue kit (Qiagen, Valencia, CA, USA) according to manufacturer's instructions. Almost 20 ml fluid sample was collected with EDTA (0.5 mol/L, pH 8.0) to reach a final concentration of 10 mmol/L EDTA. The mixture was then centrifuged at 268 g to separate the supernatant. Finally, the final supernatants of these three liquids were stored at −80°C. A peripheral blood sample of 10 ml was collected in an EDTA-containing tube for 23 patients relapsed after first-line targeted therapy. After isolated from the peripheral blood cells, plasma was subsequently frozen at −80°C. Circulating cell-free DNA (cfDNA) was purified from 4 to 5 ml of plasma using the QIAamp Circulating Nucleic Acid kit (Qiagen, Hilden, Germany).

### 2.3. NGS Library Preparation and Sequencing

NGS was carried out on all DNA samples. Sequencing libraries were prepared using a KAPA Hyper Prep kit (KAPA Biosystems, Boston, MA) with an optimized manufacturer's protocol for different samples types. In brief, 250 ng^−1^ *μ*g genomic DNA fragments or 10–250 ng cf DNA underwent end-repairing. A-tailing and ligation with indexed adapters sequentially, followed by size selection of genomic DNA using Agencourt AMPure XP beads (Beckman Coulter, Pasadena, CA). Finally, libraries were amplified by PCR and purified for target enrichment. Hybridization-based target enrichment was performed using GeneseeqOne™ 416-gene panel (Nanjing Geneseeq Technology Inc., Nanjing, China). Library fragment size was determined by an Agilent Technologies (Palo Alto, CA) 2100 Bioanalyzer. The target-enriched library was then sequenced on HiSeq4000 NGS platforms (Illumina) [13].

### 2.4. NGS Data Analysis

The raw data were aligned to Human Genome version 19 (hg19) using Torrent Suite software (version 3.6.2; Thermo Fisher Scientific, Inc.). The coverage analysis was performed using the Coverage Analysis plugin (version 3.6; Thermo Fisher Scientific, Inc.). Cases for which the quality was <20% and/or the average base coverage was <500X reads and/or the frequency was <10% were considered noninformative. Mutations were detected using the Variant Caller plugin (version 3.6; Thermo Fisher Scientific, Inc.). Each mutation was verified using the Integrative Genome Viewer (IGV) from the Broad Institute (https://www.broadinstitute.org) [14]. The NGS testing process took about five to seven days.

### 2.5. Statistical Analysis

Comparisons between groups were performed using Chi-squared test. The Kaplan-Meier method and log-rank tests were used to compare survival curves. For all the analyses, a two-sided *p* value of <0.05 was defined as significance. Statistical analyses were performed using SPSS version 16.0 (SPSS, Chicago, IL, USA).

## 3. Results

### 3.1. Patient Characteristics

As shown in Table 1, 37 (56.06%) enrolled patients were male, and 29 (43.94%) were female. The median age was 63 years old (range 33–87 years). Of all the 66 patients, 55 (83.33%) were diagnosed with lung adenocarcinoma, and 11 (16.67%) were squamous cell carcinoma. More than half (57.58%) of the patients had a history of smoking and 26 patients (39.39%) were never smokers. Another 2 cases lost the information of smoking history. There were 23 (34.85%) patients have the family history of cancer.

### 3.2. Mutation Profiling by NGS

**Figure 1:**
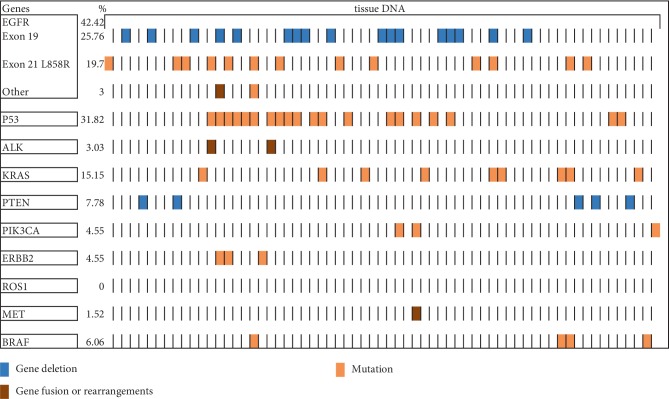
Genomic alterations detected by targeted next-generation sequencing (tNGS) in the study cohort.

A total of 66 biopsies, consisting of 60 tissue samples, three pleural effusion samples, one cerebrospinal fluid sample, and one peritoneal fluid sample were obtained from the 66 NSCLC patients. We identified 152 somatic mutations in 45 (68.18%) patients, while 21 (31.82%) patients had no somatic mutations observed. The most frequently mutated genes were *EGFR* (42.42%), *TP53* (31.82%) and *KRAS* (15.15%). Other oncogenic driver mutations including *PTEN* (7.78%), *BRAF* (6.06%), *PIK3CA* (4.55%), *ERBB2* (4.55%), and *ALK* (3.03%) were also detected among the 66 patients (Figure 1). Specifically, the most frequent *EGFR* mutation subtypes identified included exon 19 deletion (60.71%), L858R in exon 21 (46.43%). Almost all *EGFR* mutated patients carried either exon 19 or 21 mutation, and only one patient was detected to carry both mutations.

Stratified analyzed for adenocarcinoma, the most frequently mutated genes were *EGFR*(49.09%), *TP53* (34.55%) and *KRAS* (16.36%), which were similar to the full analysis sets. The *EGFR*exon 19 deletion mutation rate was higher than exon 21 L858R (30.91% versus 23.64%).

We further analyzed the information of 23 adenocarcinoma patients received the first generation of EGFR-TKI treatment after they were told *EGFR*mutated. These patients all received liquid biopsy after they relapsed or progressed. We found that 11 (47.83%) patients had mutation of *EGFR* T790M in exon 20, which was known as the most common mutation associated with acquired drug resistance. Other rare mutations included *EGFR* G719A (1.52%) and MET amplification (1.52%).

### 3.3. Clinicopathological Features and Genetic Mutations

We analyzed the association between genetic mutations and clinicopathologic variables of the enrolled patients. As shown in Table 1, *EGFR *exon 19 deletion mutation was associated with sex (*p* = 0.045), smoking history (*p* = 0.041) and family history (*p* = 0.016) and pathological type (*p* = 0.032). In another word, *EGFR *19-del mutation had an increased frequency in female, nonsmokers, who had family history and adenocarcinoma patients. However, *EGFR *L858R, *PTEN*, *ALK, *or *KRAS *were not associated with any clinicopathologic variables.

### 3.4. Utilization of NGS for Treatment Option and Prognosis Prediction in NSCLC Patients

**Figure 2:**
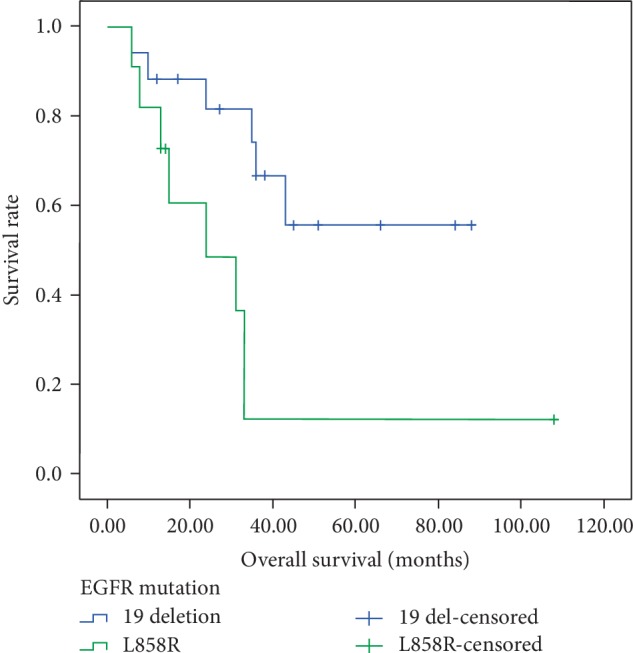
Kaplan-Meier survival curves of overall survival in NSCLC according to *EGFR* mutations.

**Figure 3:**
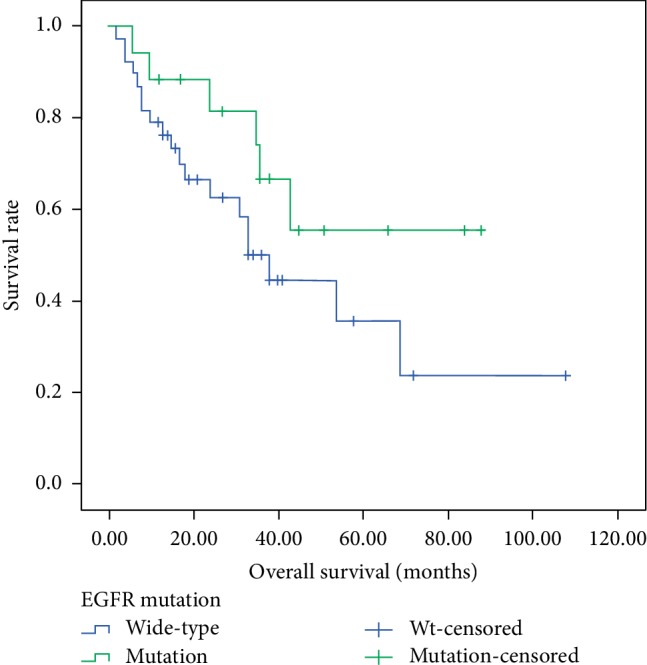
Kaplan-Meier survival curves of overall survival in adenocarcinoma according to *EGFR* 19 deletion status.

**Figure 4:**
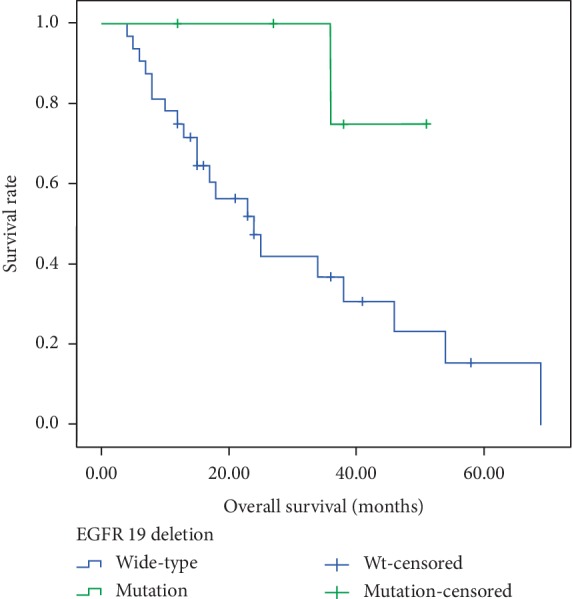
Kaplan-Meier survival curves of overall survival in smoking patients according to *EGFR* 19 deletion status.

In this study, 55 (83.33%) patients received targeted therapy such as EGFR-TKI or crizotinib after the detection of *EGFR *mutations or *ALK* gene rearrangements, and the others received chemotherapy. The median OS for target therapy patients were 24 months and the first-line PFS were 10.5 months. As shown in Figure 2, patients with *EGFR* exon 19 deletion had longer OS than those with exon 21 L858R mutation (37.0 months versus 19.0 months, *p* = 0.01). Subsequently, we further conducted stratified analysis, finding that among the adenocarcinoma cases, patients with *EGFR *19 deletion mutation have longer OS than the wide-type (36.0 months versus 19.0 months, *p* = 0.046, Figure 3). In addition, in the smoking group, patients with *EGFR *19 deletion mutation tended to have longer OS (38.0 months versus 16.5 months, *p* < 0.01, Figure 4).

**Figure 5:**
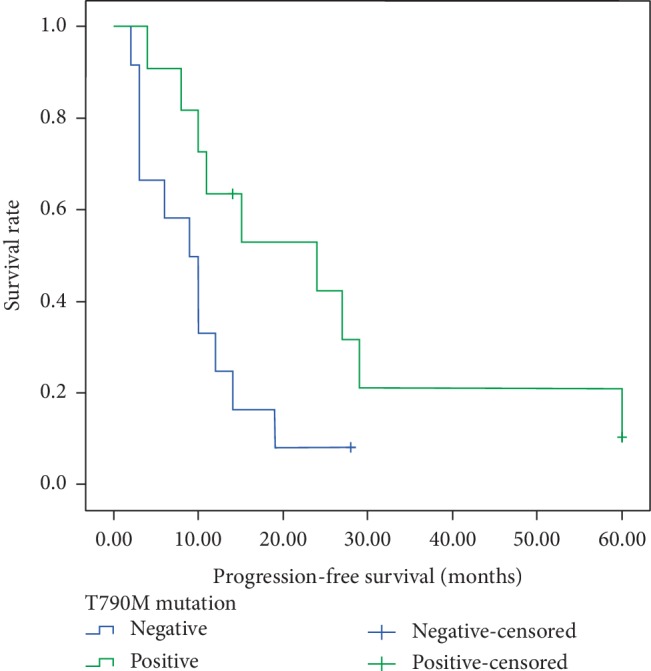
Kaplan-Meier survival curves of progression-free survival in adenocarcinoma according to *EGFR* T790M status.

After the first step of NGS detection, most of the *EGFR* mutated patients received first-generation EGFR-TKIs. The media PFS of these patients was 11 months. After disease progression, 23 patients received liquid biopsy. 11 (47.83%) patients had mutation of T790M in *EGFR* exon 20. All these 11 patients received osimertinib as the second-line targeted therapy. It was also shown that, T790M positive patients have longer PFS than the negative ones in the second-line treatment (15 months versus 9.5 months, *p* = 0.025, Figure 5).

## 4. Discussion

In the present study, we demonstrated the real-world data of mutations detected by NGS in Chinese NSCLC patients. Most of the patients benefited from the targeted therapy. We identified that NGS can be applied to guide treatment and predict prognosis in NSCLC patients.

Since its introduction in 2007, NGS technology has already made extraordinary advances, making the detection of genetic alterations to guide therapy to be more feasible. And as NGS becomes faster and less expensive, it's sure to be used more frequently and with greater benefit for Chinese NSCLC patients [15]. In our real-world data, 83.33% patients chose NGS-guided targeted therapy in first-line and/or second-line therapy. These patients, especially adenocarcinoma patients who carried driver mutations showed longer OS and PFS. Several studies have reported the use of NGS to detect the oncogenic driver mutations could guide therapy decisions and thus prolong the survival of NSCLC patients [16, 17]. We believe that our real-world data may add new evidence for it.

In our study, 23 *EGFR* mutated patients received second-line liquid biopsy after the failure of first-generation of TKI. 11 patients (47.83%) were found *EGFR* T790M mutation. All 11 patients received osimertinib therapy. Not surprising that T790M positive patients have longer PFS than the negative ones in the second-line treatment. The median OS of these patients was 36 months, which was much longer than the median 24 months of all patients. The result was consistent with the randomized phase III AURA trial that osimertinib showed a significant survival benefit in patients with advanced NSCLC who progressed to prior EGFR-TKIs and were T790M-positive [11]. We supposed that repeated NGS in relapse and metastasis NSCLC patients was essential and it also could guide the following treatment.

It was reported that, *EGFR* mutation rate varies in different countries, and the mutation rate was much higher in Asian people [18, 19]. Similar to the study conducted in southern China [20], we showed that *EGFR* was the most frequent mutation in NSCLC patients. The majority of *EGFR* mutations were exon 19 deletion (60.71%), L858R in exon 21 (46.43%). *EGFR* double mutation is not rare in Asia [21]. Analyzed for the 23 patients received second-line liquid biopsy, double mutation rate was 47.83% (11 cases), of which exon 19 deletion combined with T790M account for 81.82% (9/11). On the other hand, several studies conducted for Chinese patients reported L858R combined with T790M occurred more often [20, 22]. The different incidences of *EGFR* mutation pattern may cause by limited sample sizes and ethnic differences.

Besides *EGFR*, the mutation rate of other oncogenic driver mutations in our study such as* PTEN, KRAS*, *ALK* and *BRAF *were consistent with previous studies [23, 24]. Although driver genes mutations were reported to be mutually exclusive in NSCLC [25], we found one case carried both *EGFR L858R *mutation and* ALK* rearrangement. The patient received first-line therapy of gefitinib, and the PFS was 8 months. With one cycle of crizotinib as second-line therapy, his tumor progressed again. The OS was 15 months which was shorter than the average. This result was similar to other study. Yang et al. showed the median PFS of patients with concurrent *EGFR*/*ALK* mutations treated with EGFR-TKI ranged from 5.0 to 11.2 months, relatively lower than patients harboring only *EGFR* mutation [26].

The use of NGS in this present study revealed that females, non-smokers, family history of cancer and adenocarcinoma patients had greater *EGFR *19 deletion mutation rate, consistent with most previous reports [23, 27]. The difference of mutation rate between males and females may be caused by the higher smoking rate in males. It was also found from the stratified analysis that among the smokers, patients with *EGFR *19 deletion mutation tended to have longer OS. We think it may be due to that the mutated patients were more likely to choose the targeted therapies. Patients with EGFR exon 19 deletion were reported to have longer survival than those with exon 21 mutation [28, 29], though the detailed mechanism remain unknown. Our results added new evidences for this conclusion.

## 5. Conclusions

In summary, we applied NGS in NSCLC tumor tissue at the moment of diagnosis and in liquid biopsy at the moment of progression in a subset of *EGFR* mutant patients. It was demonstrated that using NGS in routine clinical detection allows selecting a better treatment for patients and even improving PFS and OS. Further larger-scale studies focus on the prognostic value of NGS in NSCLC and other tumors are needed to confirm the advantages of this tool.

## Figures and Tables

**Table 1:** Association between clinicopathological features and *EGFR* mutations in NSCLC patients.

Characteristics	All *n* = 66 (%)	*EGFR *19 del		*P* value	*EGFR* L858R		*P* value
		Wide-type *n* = 49 (%)	Mutant *n* = 17 (%)		Wide-type *n* = 53 (%)	Mutant *n* = 13 (%)	
*Age (years)*				0.161			0.422
≥65	29 (43.94)	24 (48.98)	5 (29.41)		22 (41.51)	7 (53.85)	
<65	37 (56.06)	25 (51.02)	12 (70.59)		31 (58.49)	6 (46.15)	

*Sex*				**0.045**			0.858
Male	37 (56.06)	31 (63.27)	6 (35.29)		30 (56.60)	7 (53.85)	
Female	29 (43.94)	18 (36.73)	11 (64.71)		23 (43.40)	6 (46.15)	

*Smoking history*				**0.041**			0.548
No	26 (39.39)	15 (30.61)	11 (64.71)		21 (39.62)	5 (38.46)	
Yes	38 (57.58)	32 (65.31)	6 (35.29)		31 (58.49)	7 (53.85)	
Unknown	2 (3.03)	2 (4.08)	0		1 (1.89)	1 (7.69)	

*Family history*				**0.016**			0.340
No	43 (65.15)	36 (73.47)	7 (41.18)		36 (22.03)	7 (53.85)	
Yes	23 (34.85)	13 (26.53)	10 (58.82)		17 (77.97)	6 (46.15)	

*Pathological type*				**0.032**			0.333
Adenocarcinoma	55 (83.33)	38 (77.55)	17 (100)		43 (81.13)	12 (92.31)	
Squamous cell carcinoma	11 (16.67)	11 (22.45)	0		10 (18.87)	1 (7.69)	

EGFR 19-del mutation had an increased frequency in female (*p* = 0.045), non-smokers(*p* = 0.041), who had family history (*p* = 0.016) and adenocarcinoma patients (*p* = 0.032).

## Data Availability

No data were used to support this study.
